# Developing an eye-tracking algorithm as a potential tool for early diagnosis of autism spectrum disorder in children

**DOI:** 10.1371/journal.pone.0188826

**Published:** 2017-11-30

**Authors:** Natalia I. Vargas-Cuentas, Avid Roman-Gonzalez, Robert H. Gilman, Franklin Barrientos, James Ting, Daniela Hidalgo, Kelly Jensen, Mirko Zimic

**Affiliations:** 1 Bioinformatics and Molecular Biology Laboratory, Research and Development Laboratory, Science and Philosophy Faculty, University Peruana Cayetano Heredia, Lima, Peru; 2 Department of International Health. School of Public Health. Johns Hopkins University, Baltimore, Maryland, United States of America; 3 School of Medicine. Johns Hopkins University, Baltimore, Maryland, United States of America; 4 Psychology Faculty. Universidad Peruana Cayetano Heredia, Lima, Peru; 5 Tulane University School of Medicine, New Orleans, LA, United States of America; Universite de Bretagne Occidentale, FRANCE

## Abstract

**Background:**

Autism spectrum disorder (ASD) currently affects nearly 1 in 160 children worldwide. In over two-thirds of evaluations, no validated diagnostics are used and gold standard diagnostic tools are used in less than 5% of evaluations. Currently, the diagnosis of ASD requires lengthy and expensive tests, in addition to clinical confirmation. Therefore, fast, cheap, portable, and easy-to-administer screening instruments for ASD are required. Several studies have shown that children with ASD have a lower preference for social scenes compared with children without ASD. Based on this, eye-tracking and measurement of gaze preference for social scenes has been used as a screening tool for ASD. Currently available eye-tracking software requires intensive calibration, training, or holding of the head to prevent interference with gaze recognition limiting its use in children with ASD.

**Methods:**

In this study, we designed a simple eye-tracking algorithm that does not require calibration or head holding, as a platform for future validation of a cost-effective ASD potential screening instrument. This system operates on a portable and inexpensive tablet to measure gaze preference of children for social compared to abstract scenes. A child watches a one-minute stimulus video composed of a social scene projected on the left side and an abstract scene projected on the right side of the tablet’s screen. We designed five stimulus videos by changing the social/abstract scenes. Every child observed all the five videos in random order. We developed an eye-tracking algorithm that calculates the child’s gaze preference for the social and abstract scenes, estimated as the percentage of the accumulated time that the child observes the left or right side of the screen, respectively. Twenty-three children without a prior history of ASD and 8 children with a clinical diagnosis of ASD were evaluated. The recorded video of the child´s eye movement was analyzed both manually by an observer and automatically by our algorithm.

**Results:**

This study demonstrates that the algorithm correctly differentiates visual preference for either the left or right side of the screen (social or abstract scenes), identifies distractions, and maintains high accuracy compared to the manual classification. The error of the algorithm was 1.52%, when compared to the gold standard of manual observation.

**Discussion:**

This tablet-based gaze preference/eye-tracking algorithm can estimate gaze preference in both children with ASD and without ASD to a high degree of accuracy, without the need for calibration, training, or restraint of the children. This system can be utilized in low-resource settings as a portable and cost-effective potential screening tool for ASD.

## Introduction

Autism spectrum disorder (ASD) currently affects nearly 1 in 160 children worldwide, resulting in an average of 111 disability adjusted life years (DALYs) lost per 100,000 people [1–5]. These children experience impaired symptoms clustered in two main categories: reduced social interactions and restricted, repetitive behavior. Disabilities in social interactions include difficulty with nonverbal behaviors like facial expression or body gestures, resulting in loss of function within social settings [6]. These individuals will often struggle in school, resulting in secondary delay in cognitive and language development [7]. Children diagnosed with ASD are not the only ones with decreased quality of life; the families of these individuals also bear the burden of social stigma and increased living expenses [8,9,10]. Recent studies have shown that early intervention for children with ASD has been effective for improving quality of life. Activities such as social conditioning have improved the individual’s ability to function in social setting [11]. In the US, every dollar spent on early intervention correlates to $8 saved in special education, crime, welfare, and other associated costs [12,13]. Despite advances in ASD diagnosis, the median age of diagnosis in the US remains at 5.5 years even though most parents begin suspecting problems around 18–24 months [14–17]. This is a result of low utilization of the gold standard diagnostic tools such as the Autism Diagnostic Interview-Revised (ADI-R) and Autism Diagnostic Observation Schedule (ADOS), even in certain high resource settings in the US [18]. The difficulty in utilizing these validated techniques can be attributed to the diagnostics’ overall length of testing (about 1.5 hours for ADOS) and extensive training for the technician (3 days with recurring retraining for ADOS) [19].

Although autism has been actively investigated in developing countries, the current prevalence and burden of ASD in many low and middle income countries (LMICs) has yet to be accurately determined. Recent research has demonstrated strong evidence for utilizing gaze preference as an early biomarker of ASD [20,21,22,23]. One study demonstrated that children with ASD showed preferential gaze fixation on the mouth, as compared to children without developmental delays [21]. Children with ASD also exhibit a preference for geometric scenes to social scenes [22,23].

Several open source eye-tracking algorithms are currently available; however these algorithms require extensive calibration and training processes that may not be followed appropriately by young children with ASD. Current eye-tracking systems for ASD diagnosis have been tested in children in controlled environments with expensive devices that require holding the head to prevent undesirable movements [22,23]. None of these solutions are appropriate in low-resource settings. Thus, a cheap, portable, and easy-to-administer tool for ASD screening is necessary for the early detection of ASD risk in low-resources rural areas.

The aim of this research is to develop and validate a portable, non-invasive, simple to use, and low-cost system based on an eye-tracking algorithm to measure gaze preference in children with and without ASD. We believe that this is the first step towards building and validating an easy-to-administer instrument to potentially screen children for ASD in low resource settings.

## Methods

### Enrollment of children

Twenty-three children between 2 and 6 years old attending a regular school in Lima, Peru, with no diagnosis or previous history of ASD were enrolled. Eight children between 2 and 6 years old attending the special center IMLA (Medical Institute of Language and Learning) in Lima, Peru, with a confirmed clinical diagnosis of ASD were also enrolled. The diagnosis of ASD for these 8 children was confirmed by a pediatric neurologist during the regular attendance of these children to the IMLA center. The diagnosis was based on a combination of clinical evaluation and previous exams including evocated auditive potentials, language and sensorial evaluations, and psychological evaluations. No ADOS-2 test was available at this time. Although there was no quantitative score for ASD available, the clinical evaluation suggested that these children were likely high-level functioning.

**Fig 1:**
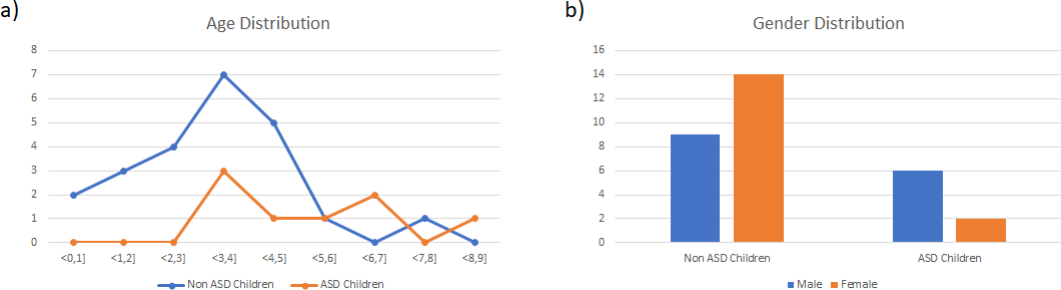
Age and gender distribution of the sample.

The mean age of participants was 48.71 months (SD 4.15) and 51.61% were females. The ratio female/male was 1.55 in the non-ASD group and 0.33 in the ASD group (Fig 1), reflecting the higher prevalence of ASD among males (Fig 1).

This study was approved by PRISMA’s Institutional Review Board (CEO921.15 April 16th, 2015). Written informed consent was obtained from parents or guardians of all children who participated in the study.

### Design of the eye-tracking system

**Fig 2:**
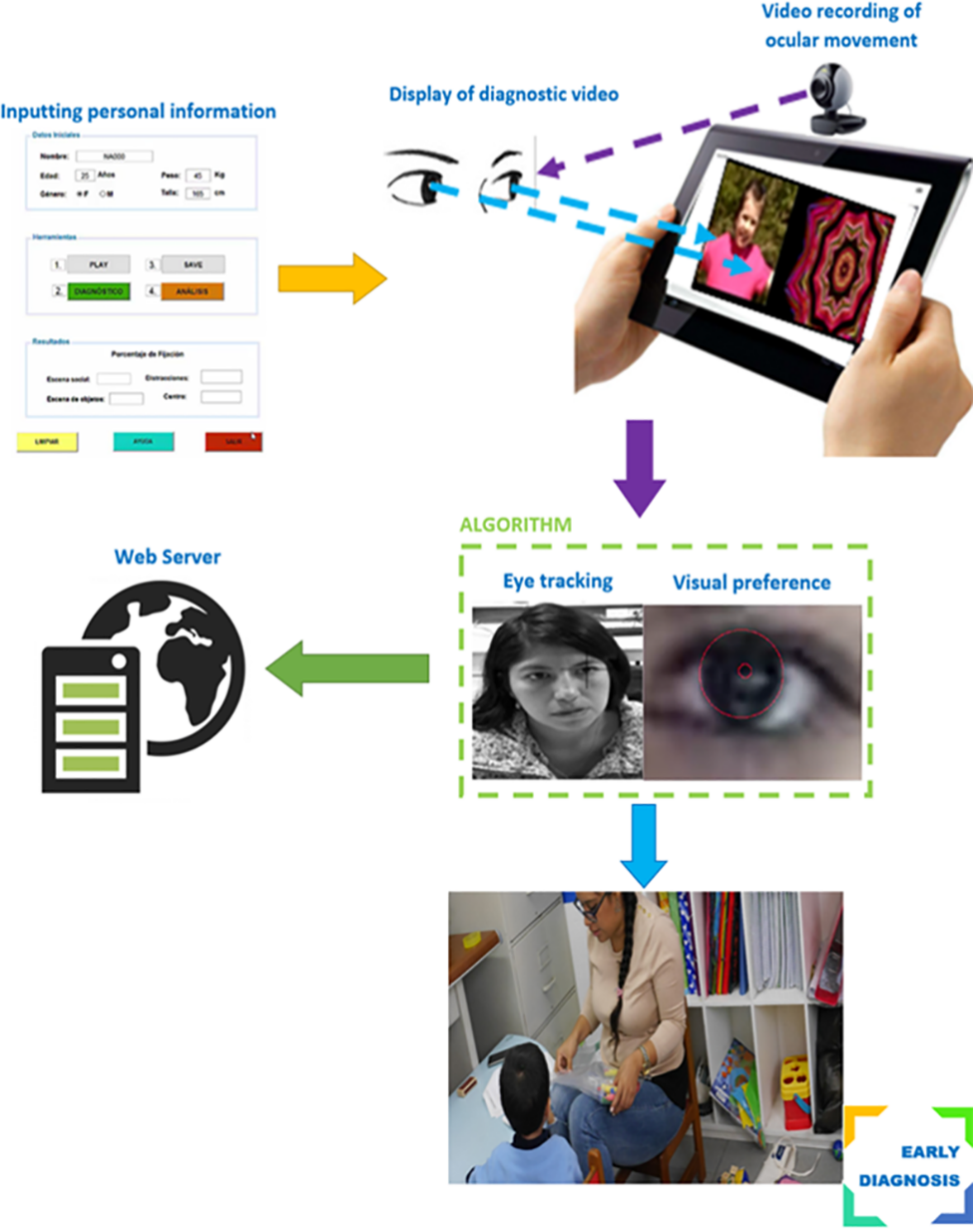
Block diagram of the eye-tracking tool illustrating the individual components.

The eye-tracking system included 5 components: (1) Inputting personal information: We developed an electronic medical record to track the patient’s performance throughout all of the appointments and to collect anonymous personal data including age, sex, weight, and height. (2) Stimulating video: A 1-minute video was created to display a social scene with playing children and an abstract scene with moving shapes on either side of the screen. (3) Recording of ocular movement: A video of the child´s face was recorded while watching the video using the tablet’s front camera. (4) Eye-tracking algorithm: An algorithm analyzed individual frames of the child’s eyes and calculated the gaze preference. (5) Data Storage: All the information collected for each child including the video of eye movements was stored on a web server for data analysis. The block diagram depicts the components of the proposed ASD diagnostic tool (Fig 2). Individuals in this manuscript has given written informed consent (as outlined in PLOS consent form) to publish these case details.

### Experimental setup

The experimental procedure was based on those described by Jones and Pierce [22,23]. A therapy room was used for tests procedures. Distractive stimuli were minimized. At the time of the test, a maximum of three people were present, the mother or guardian of the child and two study team members. The mother was instructed to sit the child in her lap and to position him/her looking directly at the camera. The child was seated at a distance of 30 cm from the tablet screen with a 90 degrees angle between the plane of the screen and the sight direction, positioned at the center of the image facing perpendicular to the tablet screen. This ensured that the child would have a detectable change in the iris position when viewing the scenes on either side of the screen. Five videos of 50 seconds each (described below) were displayed on the tablet in random order. Simultaneously, the eye movement of the child was recorded using the front camera. A total of 15,000 frames (5 videos of 50 seconds at a rate of 60 frames per second) were recorded per child for eye tracking and gaze preference analysis.

### Stimulating videos

For each video, the first ten seconds of the video included an introductory scene to capture the children’s attention. The scene included colorful, talking cartoon characters familiar to children. The scene was designed to be age-appropriate, with a non-violent positive message and popular well-known characters. In the following 50 second segment, a social scene was displayed on the right side while an abstract (non-social) scene was displayed on the left side of the screen. Both scenes were designed by a psychologist and graphic designer to make it appropriate for children less than 6 years old. These scenes were displayed with a significant amount of empty screen space in the center to ensure differences in ocular position. The social scene showed a group of children performing a physical activity, while the abstract scene displayed changing, colorful, animated non-social objects that move in front of a black background. Each video had a resolution of 1920 x 1080 pixels, 60 frames per second, and aspect ratio 16:9. For this study we used the Microsoft Surface Pro tablet based on an Intel Core i5 processor and 8GB of RAM memory.

### Eye-tracking algorithm

**Fig 3:**
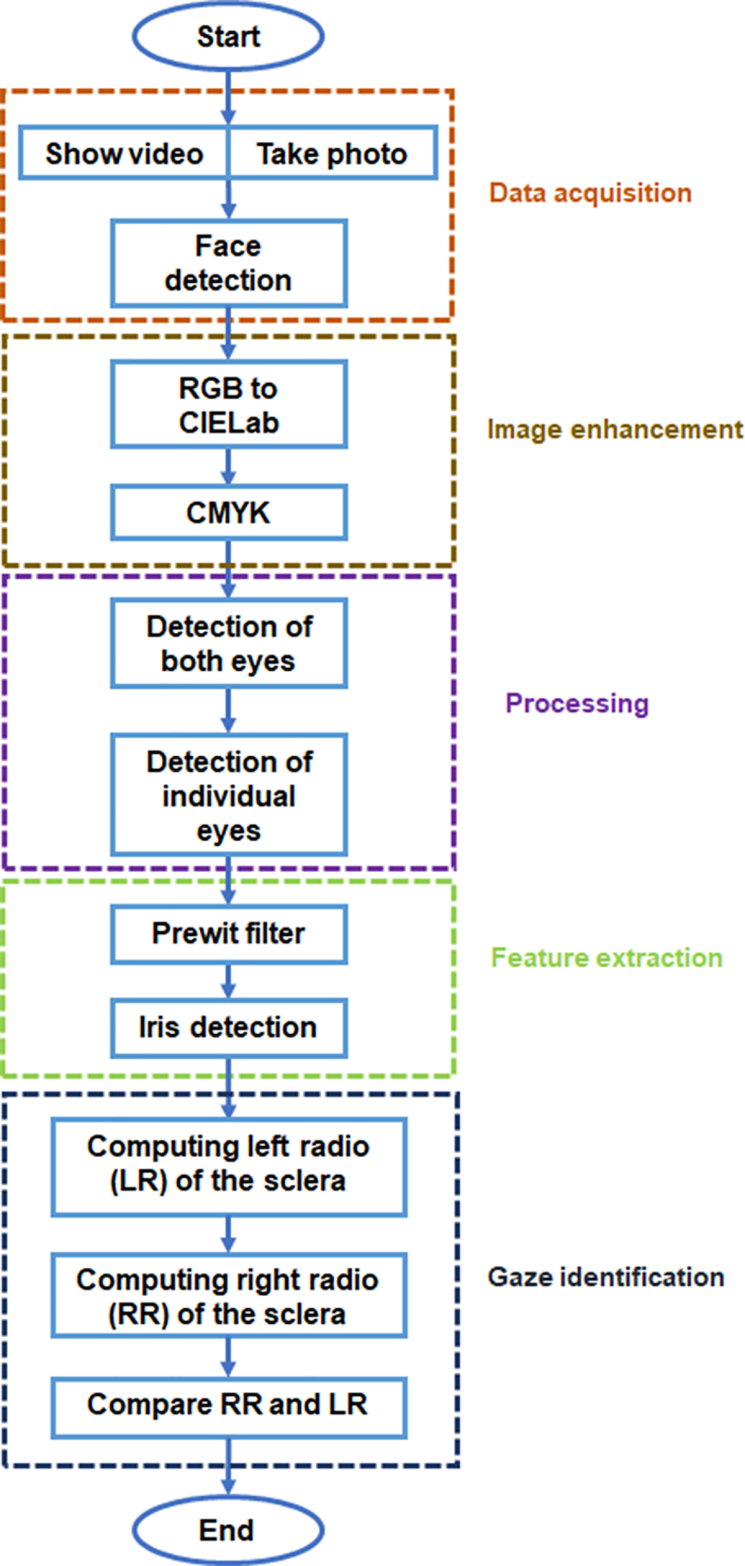
Flowchart depicting the five main stages of the algorithm designed to calculate the visual preference: 1) Data acquisition, 2) Image enhancement 3) image processing, 4) Features extraction and 5) Gaze identification.

The eye-tracking algorithm to measure gaze preference differentiated between four different cases of visual gaze for each individual frame. Right: if both eyes had a right gaze. Left: If both eyes had a left gaze. Distraction: if both eyes were not simultaneously detected. This case registered as a distraction because the child was drawn to neither the social nor the abstract scene. Center: if both eyes had a central gaze. It is important to note that this specific situation qualifies neither as a left gaze or right gaze; thus the child is looking at neither the social nor the abstract scene. The algorithm consisted of 5 main processes: data acquisition, image enhancement, processing, features extraction, and gaze identification (Fig 3).

#### Data acquisition

Obtaining the image: In this step we projected the stimulating video onto the tablet and simultaneously recorded the child with the webcam. We then divided the video obtained into individual frames, obtaining 1800 frames on average. We were at times unable to obtain all 3000 frames for the 50 seconds because the child was unwilling to cooperate for the entirety of the video.

**Fig 4:**
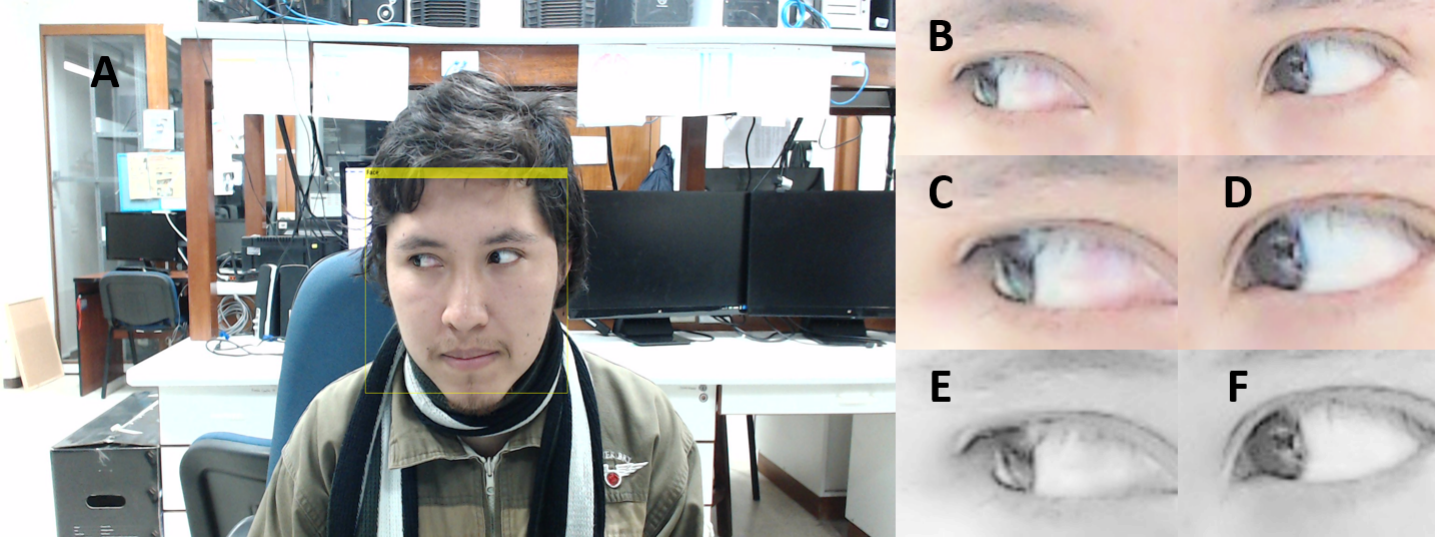
Detection of (A) face, (B) both eyes, and (C) individual right eye. (D) Individual left eye. (E) grayscale conversion, and (F) contrast enhancement are performed.

Face detection: Detection of the face was completed using the Viola-Jones algorithm [24], by utilizing weak classifiers based on a decision stump provided in MATLAB. These classifiers utilized Haar features to encode the details of the face [24]. We performed this process first to optimize the processing time of the algorithm (Fig 4A).

#### Image enhancement

After performing the facial recognition step, it was necessary to improve the image obtained. We converted RGB values of the image to CIELab values. This color space describes mathematically all perceivable colors in the three dimension *L* for lightness and *a* and *b* for the color opponents green–red and blue–yellow. Subsequently we adjusted the lightness contrast using the *L* component, converting the cluster into an histogram. This restrained most pixels in the *L* component to a new range of values between 0–255 in gray-scale, with a gamma equal to 0.5, where gamma specifies the shape of the curve describing the relationship between the values in I and J which are the initial range of values of the L component and the new range of values. Once we completed the last step we developed the same procedure with CMYK color space using a gamma value of 3 to achieve enhanced black regions of the image.

#### Processing

Two eye detection: This was performed in a series of cascade classifications based on a decision tree, starting with the detection of the face, followed by the identification of two eyes. If the face detection was not carried out then we continued to the next step. The Viola-Jones algorithm with weak classifiers provided by MATLAB was again utilized to detect both eyes (Fig 4B). Detection of the individual eyes was performed with the Viola-Jones algorithm with weak classifiers.

Single eye detection: Detection of each individual eye was performed, as the last step in a series of cascade classifications based on a decision tree, to identify the right eye and the left eye (Fig 4C and 4D).

#### Features extraction

In this step we detected the border of the eyes and the position of the iris. A Prewit filter was used for feature enhancement allowing detection of the iris. Furthermore, to enhance the region containing the sclera (Fig 4E and 4F), we converted RGB values of the image to CIELab values and we obtained a horizontal vector from the L component. This horizontal vector was located in the iris’s center with a radius greater than iris’s radius.

After we analyzed the vector’s brightness profile, this procedure gave us information about the iris and the sclera with small and large values respectively. The change in the brightness level between the regions belonging to the sclera, (taking into account that the iris is located in the middle of the sclera), gave us the patient’s gaze orientation.

#### Gaze identification

**Fig 5:**
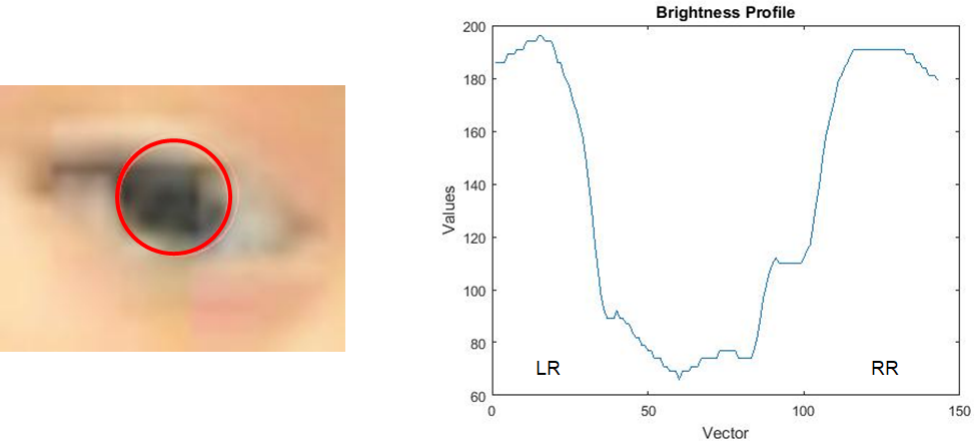
Iris detected image and brightness profile of the horizontal vector.

The orientation of the gaze was determined by the radius between the brightest pixels belonging to each region of the sclera, where one represents the left region (LR) and the other represents the right region of the sclera (RR) (Fig 5). If the LR/RR radius was less than or equal to the threshold value, this meant that the child's gaze was focused on the right side of the screen and toward the dynamic object scene. If the RR/LR radius was less than or equal to the threshold value, this indicated that the child was focused in the left side of the screen, towards the social scene. In the case of a central gaze, if the radius of the minimum and the maximum between the values of LR and RR was greater than the threshold value, it suggested that the orientation of the child's gaze was focused on the middle of the screen.

The values established for the threshold and other parameters that composed the algorithm were determined by a test that maximized the concordance in the classification of the categories Left and Right, based on manually classified images. In total, 18 models were evaluated, of which, as mentioned, the model with the highest degree of agreement with the manual classification was selected. This model established a threshold equal to 0.85 to determine the orientation of the gaze in the corresponding images of patients.

### Graphical user interface

**Fig 6:**
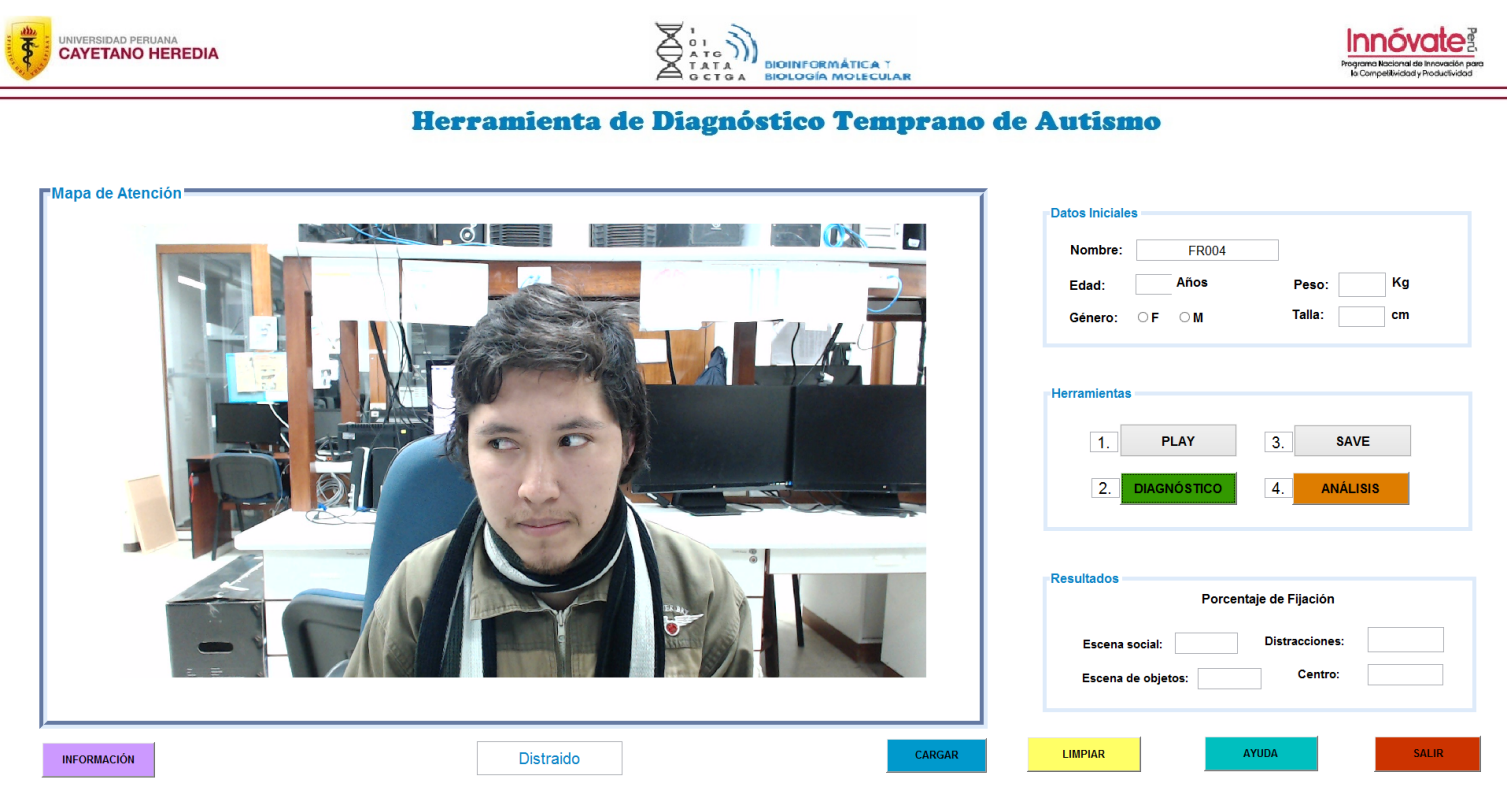
Graphical user interface of the eye-tracking tool, demonstrating the patient data input, the main controls for the video display and video capture, and visual preference data output. Demonstration of the frame-by-frame analysis for visual preference by displaying pupil position for both eyes, allowing for investigator corroboration.

A graphical user interface (GUI) was created to display the available actions of the algorithm allowing a simple execution of the diagnostic tool. The GUI incorporated a section for entering the personal data of the child, as well as buttons for displaying the stimulating video (PLAY), saving the video of the child (SAVE), beginning the data analysis of the recorded video (DIAGNOSTIC), and displaying a historical evolution curve of gaze preference results for the individual patient being analyzed (ANALYSIS), (Fig 6). In the last section, the calculated results with the percentages of the gaze fixation are displayed.

### Validation of the eye-tracking algorithm

The eye tracking and gaze preference analysis were conducted both automatically and manually. The algorithm analyzed the 5 videos using the procedures described above to classify each frame independently and calculated the percentages of time the children spent looking at the social scene or abstract scene, or were distracted. For comparison, one human observer manually analyzed the 5 videos frame by frame. Each frame was observed and manually classified every 15 frames to determine the position to which the child was looking (left or right) or if there was a distraction.

The manual and the automatic classification (left or right) of the corresponding frames were compared. For this, based on a frame-level, the Spearman correlation was calculated.

Given that for screening purposes, the gaze preference towards a specific scene is of ultimate importance, we compared the gaze preference to a particular scene during the entire period of evaluation. The gaze preference was estimated as the percentage of the time looking at a particular scene for the total five videos. Gaze preferences estimated by manual measurements and by the automatic analysis performed by the eye-tracking algorithm, were compared using a paired student’s T-test. For these comparisons, only the “left” and “right” categories were used.

In addition, and as an exploratory analysis only, the percentage of time looking at the social/abstract scenes (measured manually and automatically by the algorithm), were compared between the children with ASD and the non-ASD group using a T-test for each stimulating video. The statistical analysis was performed with 5% significance using Stata 14.2 (STATACorp, College Station, Texas, USA). The complete dataset of this study is available in S1 Table.

## Results

**Fig 7:**
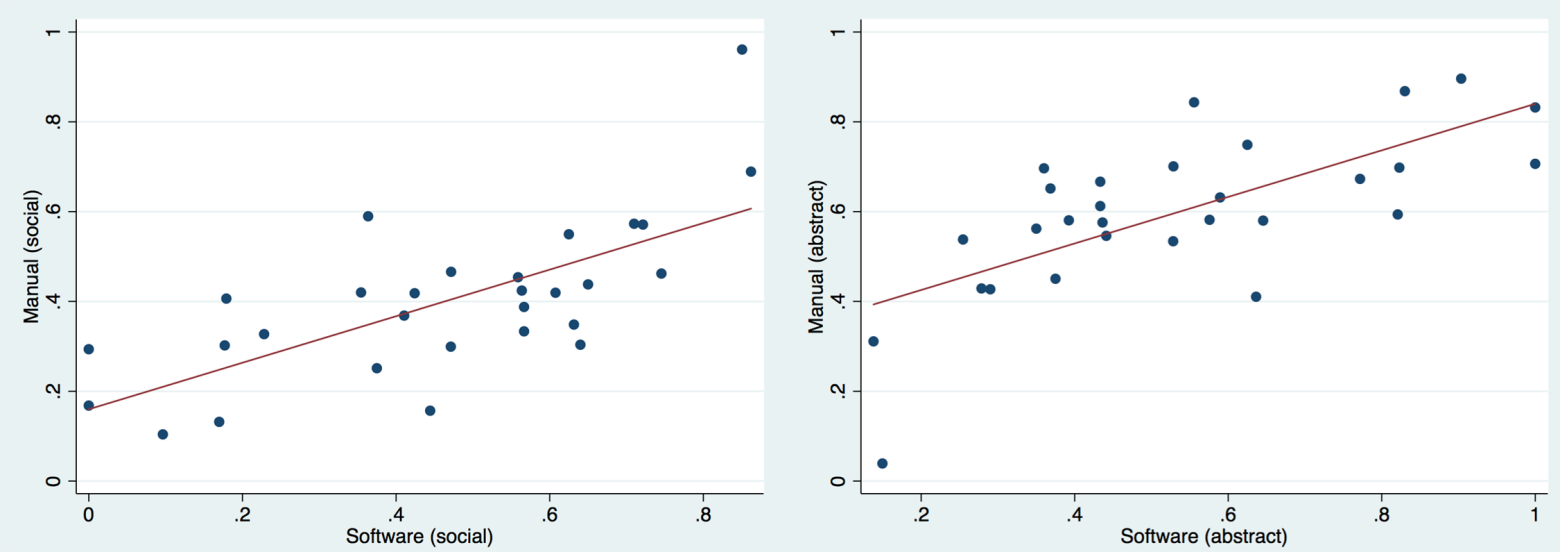
Correlation / scatter plot between the manual and algorithm estimates for the percentages of time of gaze preference towards the abstract/social scenes and distractions.

The main result confirmed the validity of the algorithm to correctly classify gaze preference at a frame level. After removing the frames classified as distractions from both the manual and the automatic analysis, and keeping only the left/right (social/abstract scenes) frames, and by considering the 5 videos, the correlation between the manual and the automatic classifications for left/right gaze, was 73.24% (Spearman statistic, P<0.0001). When comparing the percentage of time observing to the right (or to the left), estimated by the manual analysis versus the automatic analysis, the correlation was 70% (Pearson statistic, P<0.0001) (Fig 7).

The mean percentage of time observing the abstract scene estimated manually (56.11%) was not significantly different from the time estimated automatically by the algorithm (54.59%), (difference of 1.52%, equivalent to 0.75 seconds, P = 0.136 for a proportion test), (Table 1). Similarly, there was no significant difference between the estimates by the observer and the algorithm for the social scene (difference of 1.53% equivalent to 0.75 seconds, P = 0.136 for a proportion test), (Table 1).

**Table 1:** Manual and automatic (performed by the algorithm) evaluation of the percentage of time preference towards the social and abstract scenes. No significant difference is observed between the two methods.

N = 2,559 frames		Manual evaluation	Software evaluation	P-value
Social scene	Mean (SE) of the percentage of time preference	1,123/2,559 43.88% (0.98%)	1,162/2,559 45.41% (0.98%)	0.136
	95% CI	[41.95%, 45.83%]	[43.46%, 47.36%]	
Abstract scene	Mean (SE) of the % of time preference	1,436/2,559 56.11% (0.98%)	1,397/2,559 54.59% (0.98%)	0.136
	95% CI	[54.16%, 58.04%]	[52.63%, 56.53%]	

When considering the manual evaluation, in average, children with ASD spent 26.96% of the time viewing the social scene while children without ASD spent a significantly higher amount of time, (44.21%) watching the social scene (P = 0.01). Based on the algorithm evaluation, children with ASD spent 32.86% of the time viewing the social scene while children without ASD spent a significantly higher amount of time (50.74%) (P = 0.04), (Table 2).

**Table 2:** Comparison of gaze preference towards the social scene between children with ASD and children with no ASD, according to the manual and software estimations.

			Children with ASD (N = 8)	Children without ASD (N = 23)	P-value
Manual Evaluation	Social scene	Mean (SE) of the % of time preference	26.96 (6.07)	44.21 (3.49)	0.01
		95% CI	[12.09, 41.83]	[36.94, 51.49]	
Software Evaluation	Social scene	Mean (SE) of the % of time preference	32.86 (10.48)	50.74 (4.57)	0.04
		95% CI	[7.20, 58.52]	[41.22, 60.27]	

## Discussion

This study demonstrated an eye-tracking algorithm using a tablet-based system estimates gaze preference children with and without ASD with sufficient accuracy. It is important to highlight that this system works without requiring intensive calibration, previous training, or restriction of the child’s head movement making it applicable in the ASD population including young children.

We developed an algorithm that would facilitate the use of a portable eye-tracking diagnostic system and eliminate the need for manual analysis. The results suggested that an algorithm, while not a perfect substitute for manual analysis of the videos, produced similar results. The current design of the algorithm can be run as an executable application on a tablet. The program requires a tablet or computer with a built in or mounted front camera but does not require a computer or tablet with high processing power. For local machines that cannot handle the processing needed to calculate the visual preference at the site of diagnosis, the program can be used to capture video data. It can then send the video through an Internet connection to a site equipped to handle the computing demands, and receive the processed results making it more cost-effective than previously used methods. Additionally, the program is both portable and easy to use requiring minimal training in execution of the visual stimulus, video recording, and running the data analysis program.

There were a number of limitations in this study. We did not compare the importance of the positioning of the social and abstract scenes and therefore do not know if placement on the left or right side of the scene is related to visual preference. Further studies are required to understand this issue. Additionally, the algorithm we developed analyzed the video frame-by-frame. To validate the algorithm, we compared its results with a frame-by-frame manual analysis using one of every 15 frames. Although it is possible for a human observer to verify each of the 30,000 frames categorized by the algorithm, this seemed unnecessary. The differences between the algorithm results and the manual results may be due to non-exact human measurements.

Because our algorithm only looks at the relative position of the pupil within the eye, it may detect if the children are looking to their left or right but does not always accurately detect the exact object of their gaze. The child may have had a head position so far removed from the center that a left-ward gaze towards the right-sided scene will be misinterpreted by the algorithm as a left-ward gaze towards the left-sided scene. Additionally, our algorithm does not have the capacity to identify distractions towards objects that exist above or below our tablet screen. For example, if a toy is located beyond the tablet screen on the child's left side, the algorithm will register this as a left-ward gaze instead of as a distraction.

This was a small study and larger case-control studies will be important for validation of this new potential screening tool. Our small pilot study noted a statistically significant difference in age and gender between the ASD-diagnosed children and non-ASD children. Thus, larger sample sizes may elucidate the confounding factors and true correlations. Future testing of the sensitivity and specificity of such a diagnostic tool with different design parameters (specific introductory scenes, social scenes, abstract scenes, and GUI layout) should be studied to determine the specific efficacy of such a tool.

While the preference for scenes with objects rather than social scenes in autistic children has been described previously, its use as a screening tool has been limited. These previous studies used either bulky equipment that required physical constraints or expensive computer equipment [22, 23]. In this study, we sought to develop an algorithm that would be the basis for a cost-effective and portable screening tool based on previous research utilizing visual preference as an earlier marker for ASD. The development of this tool required selection of appropriate stimulating videos, creation of an algorithm, and validation of the application as a tool to estimate gaze preference. To maximize the accuracy of our test, we chose dynamic social and dynamic abstract scenes that did not overlap in content.

One of the first studies to look at visual preferences in ASD as a diagnostic tool was done by Karen Pierce in 2011. This study reported a preference for geometric scenes in children with ASD [23]. Similar to this study by Pierce, we used a video of children performing visual activities as the dynamic social scene and colorful moving objects as the dynamic abstract scene.

Based on these previous studies, we developed an eye-tracking system that uses minimal resources making it both portable and cost-effective for ASD screening in the future. We hope to utilize the algorithm in the development of an ideal tool for early autism screening in LMIC’s. While follow-up studies are needed to test the sensitivity and specificity of the tablet application, we have shown that an algorithm can be used to analyze the eye-tracking videos. This has important implications for increasing the availability of autism screening especially in areas without trained personnel in gold-standard methods to diagnose autism and other resource intensive diagnostic techniques. Visual preference studies have demonstrated promise in the early detection of autism and the implementation of a cost-effective system may increase early diagnosis thus enabling early intervention and treatment delivery.

## Supporting information

S1 Table Data set. (XLSX)
